# Suppression of cell wall-related genes associated with stunting of *Oryza glaberrima* infected with *Rice tungro spherical virus*

**DOI:** 10.3389/fmicb.2014.00026

**Published:** 2014-02-04

**Authors:** Bernard O. Budot, Jaymee R. Encabo, Israel Dave V. Ambita, Genelou A. Atienza-Grande, Kouji Satoh, Hiroaki Kondoh, Victor J. Ulat, Ramil Mauleon, Shoshi Kikuchi, Il-Ryong Choi

**Affiliations:** ^1^Plant Breeding, Genetics, and Biotechnology Division, International Rice Research InstituteMetro Manila, Philippines; ^2^Plant Genome Research Unit, Agrogenomics Research Center, National Institute of Agrobiological SciencesTsukuba, Ibaraki, Japan; ^3^T. T. Chang Genetic Resources Center, International Rice Research InstituteMetro Manila, Philippines

**Keywords:** *Oryza glaberrima*, *Rice tungro spherical virus*, stunting, cell wall genes

## Abstract

Rice tungro disease is a complex disease caused by the interaction between *Rice tungro bacilliform virus* and *Rice tungro spherical virus* (RTSV). RTSV alone does not cause recognizable symptoms in most Asian rice (*Oryza sativa*) plants, whereas some African rice (*O. glaberrima*) plants were found to become stunted by RTSV. Stunting of rice plants by virus infections usually accompanies the suppression of various cell wall-related genes. The expression of cell wall-related genes was examined in *O. glaberrima* and *O. sativa* infected with RTSV to see the relationship between the severity of stunting and the suppression of cell wall-related genes by RTSV. The heights of four accessions of *O. glaberrima* were found to decline by 14–34% at 28 days post-inoculation (dpi) with RTSV, whereas the height reduction of *O. sativa* plants by RTSV was not significant. RTSV accumulated more in *O. glaberrima* plants than in *O. sativa* plants, but the level of RTSV accumulation was not correlated with the degree of height reduction among the four accessions of *O. glaberrima*. Examination for expression of genes for cellulose synthase A5 (CESA5) and A6 (CESA6), cellulose synthase-like A9 (CSLA9) and C7, and α-expansin 1 (expansin 1) and 15 precursors in *O. glaberrima* and *O. sativa* plants between 7 and 28 dpi with RTSV showed that the genes such as those for CESA5, CESA6, CSLA9, and expansin 1were more significantly suppressed in stunted plants of *O. glaberrima* at 14 dpi with RTSV than in *O. sativa*, suggesting that stunting of *O. glaberrima* might be associated with these cell wall-related genes suppressed by RTSV. Examination for expression of these genes in *O. sativa* plants infected with other rice viruses in previous studies indicated that the suppression of the expansin 1 gene is likely to be a signature response commonly associated with virus-induced stunting of *Oryza* species. These results suggest that stunting of *O. glaberrima* by RTSV infection might be associated with the suppression of these cell wall-related genes at the early stage of infection with RTSV.

## Introduction

Rice tungro disease (RTD) is one of the major constraints to rice production in South and Southeast Asia (Azzam and Chancellor, 2002). RTD is a composite disease caused by two taxonomically unrelated viruses, *Rice tungro bacilliform virus* (RTBV) and *Rice tungro spherical virus* (RTSV) both transmitted by green leafhoppers (GLH) (Hull, 1996). RTBV is a plant pararetrovirus belonging to the family *Caulimoviridae*, genus *Tungrovirus*, with a circular, double-stranded DNA genome encapsidated in bacilliform particles (Fauquet et al., 2005). RTSV is the type member of the *Sequiviridae* family, genus *Waikavirus*, having a single-stranded polyadenylated plus-sense RNA genome encapsidated in polyhedral particles (Choi, 2008). Asian rice (*Oryza sativa*) plants infected with both RTBV and RTSV usually show symptoms such as severe stunting, yellowing of the leaves, and reduced tillering (Azzam and Chancellor, 2002). *O. sativa* plants infected with RTBV alone exhibit recognizable stunting and yellowing of the leaves, whereas *O. sativa* plants infected with RTSV alone exhibit very mild stunting or no clear symptoms (Hibino et al., 1990).

African rice (*O. glaberrima*) is known to have many favorable traits such as weed competitiveness, drought tolerance, pest resistance, and the ability to grow under low-input conditions (Sarla and Swamy, 2005, and references therein). Efforts have been made to transfer genes conferring such useful traits from *O. glaberrima* to *O. sativa* through inter-specific hybridization (Sarla and Swamy, 2005, and references therein). However, some accessions of *O. glaberrima* were found to be hypersensitive to RTD, and to be significantly stunted even when infected with RTSV alone (Cabauatan et al., 1993).

Stunting is a common symptom of *O. sativa* infected with viruses such as *Rice dwarf virus* (RDV), *Rice grassy stunt virus, Rice ragged stunt virus, Rice stripe virus* (RSV), and RTBV (Hibino, 1996). Genome-wide gene expression analyses in *O. sativa* plants infected with RDV (Shimizu et al., 2007; Satoh et al., 2011) and RSV (Satoh et al., 2010) revealed that many stress response-related genes were activated, whereas various development-related genes, including genes involved in cell wall synthesis, were suppressed in *O. sativa* plants infected with RDV and RSV, indicating that stunting of *O. sativa* plants by RDV and by RSV might be associated with the suppression of various development-related genes, especially cell wall-related genes. The expression of many defense- and development-related genes, including cell wall-related genes was also found to be regulated in *O. sativa* plants by infection with RTSV (Satoh et al., 2013). However, the consequences from such changes in gene expression by RTSV were uncertain since the *O. sativa* plants infected with RTSV remained asymptomatic.

In this study, we examined the relationship between the severity of stunting and the expression of six cell wall-related genes in *O. glaberrima* infected with RTSV to see whether stunting of *O. glaberrima* is associated with the capability (pathogenicity) of RTSV to suppress the expression of cell wall-related genes. The results showed that the cell wall-related genes such as those for α-expansin 1 precursor (expansin 1), cellulose synthase A5 (CESA5), cellulose synthase A6 (CESA6), and cellulose synthase-like A9 (CSLA9) were more significantly suppressed in stunted plants of *O. glaberrima* at the early stage of infection with RTSV than in *O. sativa*, suggesting that the suppression of these genes at the early infection stage is signature responses associated with stunting of *O. glaberrima* induced by RTSV infection.

## Materials and methods

### Plant materials

Eighteen accessions of *O. glaberrima* (International Rice Germplasm Collection (IRGC) accession numbers 86741, 96717, 96718, 96790, 96793, 96864, 96868, 100139, 100153, 102556, 102569, 103437, 103477, 104545, 104589, 104914, 112576, and 115633) were obtained from the T. T. Chang Genetic Resources Center, International Rice Research Institute (IRRI). *O. sativa* cultivar Taichung Native 1 (TN1) is susceptible to RTSV. TW16 is a backcross line (BC_5_) resistant to RTSV, and was developed by serial backcrosses of RTSV-resistant cultivar Utri Merah (IRGC number 16682) with TN1 (Lee et al., 2010).

### Inoculation of RTSV

RTSV strain A (Cabauatan et al., 1995) maintained in TN1 was used as the source of inoculum. Five plants each of TN1 and TW16, and 18 *O. glaberrima* accessions were used per treatment (mock control or RTSV-inoculated) and sampling timing [7, 14, 21, 28, or 30 days post-inoculation (dpi)]. The plants were grown in a 12-cm-diameter pot with each pot containing five seedlings. GLH-mediated inoculation of RTSV to plants was done by the tube method as described by Cabauatan et al. (1995). GLH were given a 3-day acquisition access period to RTSV-infected plants and were allowed an inoculation access period of 24 h to 9-day-old plants at three insects per plant. Plants for mock control were prepared by feeding three virus-free insects per plant for 24 h. At Twenty Four hours after inoculation started, GLH were removed by insecticide. The inoculated plants were maintained in the greenhouse of IRRI.

### Evaluation for reactions to RTSV

The height of individual mock- and RTSV-inoculated plants was measured at 7, 14, 21, and 28 dpi) (*n* = 5 per treatment and time point in one experiment). Height reduction rates (%) due to RTSV infection were computed as 100 × [(height of mock-inoculated plant—height of RTSV-infected plant)/height of mock-inoculated plant]. After the height measurement, the second youngest fully expanded leaf was collected from each plant for RNA extraction (see below). The leaf samples were quickly frozen in liquid nitrogen and immediately kept at −80°C until RNA extraction. Subsequently, approximately 1/3 of the upper part of each plant was collected for evaluation of RTSV accumulation in the plant. One gram of plant samples collected were pulverized in liquid nitrogen, and homogenized with 10 ml of phosphate buffer saline containing Tween-20 to prepare 10 time-diluted sap samples. The diluted plant sap samples were used to estimate RTSV accumulation in plants by enzyme-linked immunosorbent assay (ELISA) using an antibody raised against purified RTSV as described by Shibata et al. (2007). The experiment for height measurement and that for estimation of RTSV accumulation were repeated three times. The differences between the heights of mock- and RTSV-inoculated plants were examined by the analysis of variance (ANOVA) and a *t*-test, and the differences in height reduction rate and RTSV accumulation (absorbance at 405 nm in ELISA) among *O. glaberrima* and *O. sativa* plants were examined by ANOVA and the least significant difference (LSD) test.

### Selection of cell wall-related genes for expression analysis

Cell wall-related genes investigated for expression in *O. glaberrima* (Table 1) were selected based on their expression patterns in *O. sativa* TN1 and TW16 after infection with RTSV (Satoh et al., 2013). Genes orthologous between *O. sativa* and *O. glaberrima* were identified by BLAST-Like Alignment Tool (BLAT) (Kent, 2002) using the *O. sativa* japonica gene sequences (http://rice.plantbiology.msu.edu) as query and the *O. glaberrima* genome assembly (www.genome.arizona.edu) as the database. Genes were considered to be orthologous if the length of the BLAT match along the *O. glaberrima* scaffold is more than 90% of the length of the *O. sativa* japonica gene with minimal mismatches.

**Table 1:** **Cell wall-related genes examined for expression in *Oryza glaberrima* and *O. sativa* infected with *Rice tungro spherical virus***.

**Genes for**	**Locus ID in *O. sativa***	***O. glaberrima s*caffold ID**	**BLAT match (%)**	**Primers for RT-PCR (5′–3′)**		**Expected amplicon size (bp)**
Cellulose synthase A5 (CESA5)	LOC_Os03g62090	Oglab03_0146	99.6	Forward	ATCTGCCGTCTGGAATAGAG	305
				Reverse	ACCCACTCATCTCCAGTGTT	
Cellulose synthase A6 (CESA6)	LOC_Os07g14850	Oglab07_0051	99.8	Forward	CCATATATGCGGTGATACGA	344
				Reverse	CTTCCTTCCTTCTGTCCAAA	
Cellulose synthase-like A9 (CSLA9)	LOC_Os06g42020	Oglab06_0208	99.3	Forward	GTGTGCAAGTGCAAAGTTTC	304
				Reverse	GGTGAGCATATTGAGGAACC	
Cellulose synthase-like C7 (CSLC7)	LOC_Os05g43530	Oglab05_0141	98.5	Forward	GAAAACACTTTGCAAGCATC	303
				Reverse	ACCCAAGGTAATCGATCAAA	
α-Expansin 1 precursor	LOC_Os05g39990	Oglab05_0133	99.2	Forward	GTGTCCTTCTTCGTCTTCGT	447
				Reverse	ACTCCTCCATTACACCCAAA	
α-Expansin 15 precursor	LOC_Os02g51040	Oglab02_0356	99.3	Forward	CGTCGTGGTAGTTGCAGTAG	468
				Reverse	TGCATTAATACTCCCGCATA	
Actin	LOC_Os03g50885	Oglab03_unplaced058	99.5	Forward	TCCATCTTGGCATCTCTCAG	337
				Reverse	CAGATGCCTGATGAGGGTAC	

### Real-time RT-PCR for cell wall-related genes

Total RNA was isolated individually from the second fully expanded leaves of single plants using TRIzol (Invitrogen, USA) according to the manufacturer's instructions. The equal amounts of the RNA samples from five independent plants of the same treatment were pooled prior to cDNA synthesis. First-strand cDNA molecules were synthesized from 3 μg of the pooled RNA samples using SuperScript III (Invitrogen, USA) and an oligo(dT) primer (Invitrogen, USA) in 20 μl of reaction mix according to the manufacturer's instruction. Primers specific to six cell wall-related genes were designed to target the 3′ untranslated regions of the corresponding orthologs in both *O. sativa* and *O. glaberrima*, and to avoid amplification from the gene family of selected genes (Table 1). Real-time PCR was performed with the gene-specific primers using the Light Cycler 480 SYBR Green I Master Mix (Roche, USA) in a Light Cycler 480 thermal cycler (Roche, USA) according to the manufacturer's instructions. Three replicates were performed for each sample. A threshold cycle (C_*T*_) value was obtained for each reaction. Fold changes in cell wall-related gene expression between mock-inoculated and RTSV-inoculated plants were computed using the comparative C_*T*_ method (Schmittgen et al., 2000) with an actin gene (LOC_Os03g50885 in *O. sativa* and Oglab03_unplaced058 in *O. glaberrima*) as the internal reference control (Table 1). The differences in fold changes in expression of the genes among *O. sativa* and *O. glaberrima* plants were examined by ANOVA and LSD test.

## Results

### Stunting of *O. glaberrima* by RTSV

Eighteen accessions of *O. glaberrima* were inoculated with RTSV to see whether RTSV induces stunting in *O. glaberrima* accessions. Most accessions of *O. glaberrima* infected with RTSV appeared to be evidently stunted at 30 dpi (Supplementary Material 1), and the number of tillers usually decreased in the plants stunted with RTSV. However, many plants of *O. glaberrima* accessions died or grew poorly during the initial evaluation. This was probably due to a loss of vigor after long-term storage. Therefore, among the 18 accessions of *O. glaberrima*, we selected and propagated four (96790, 96793, 102569, and 104545) for further evaluation to confirm their reactions to RTSV. They were selected because *O. glaberrima*-96793 and -102569 appeared to be severely stunted by RTSV infection in the preliminary evaluation, whereas *O. glaberrima*-96790 and -104545 seemed not stunted or stunted only slightly.

The height reduction due to RTSV infection in the four accessions of *O. glaberrima* and two genotypes of *O. sativa*, TN1 susceptible to RTSV and TW16 resistant to RTSV, was monitored until 28 dpi to characterize the stunting phenotypes of the plants. The heights of *O. sativa* TN1 and TW16 were not reduced significantly after infection with RTSV, whereas the heights of the four accessions of *O. glaberrima* were significantly reduced after RTSV infection (Table 2).

**Table 2:** **Heights of mock—and *Rice tungro spherical virus* (RTSV)-inoculated plants of *Oryza sativa* and *O. glaberrima***.

**Plant**	**Treatment**	**Plant height^1^ (cm) at**			
		**7 dpi**	**14 dpi**	**21 dpi**	**28 dpi**
*O. sativa*—TW16	Mock	27.9 ± 1.0	44.1 ± 1.0	55.4 ± 0.9	69.9 ± 1.4
	RTSV	27.2 ± 1.3	43.2 ± 1.2	55.5 ± 0.8	69.8 ± 1.6
*O. sativa*—TN1	Mock	28.3 ± 0.9	43.0 ± 0.9	54.4 ± 0.7	68.1 ± 1.7
	RTSV	26.9 ± 0.9	41.2 ± 1.0	52.2 ± 1.1	66.6 ± 2.0
*O. glaberrima*—96790	Mock	28.0 ± 1.0	50.6 ± 0.5a	69.4 ± 1.6a	88.0 ± 2.2a
	RTSV	25.7 ± 1.1	41.2 ± 1.5b	57.4 ± 2.1b	75.0 ± 2.6b
*O. glaberrima*—96793	Mock	28.1 ± 1.1a	52.3 ± 1.0a	74.4 ± 0.9a	94.9 ± 2.3a
	RTSV	24.9 ± 0.8b	42.8 ± 1.8b	61.3 ± 1.2b	81.6 ± 1.4b
*O. glaberrima*—102569	Mock	23.6 ± 0.7a	42.0 ± 1.4a	64.1 ± 1.6a	82.0 ± 2.5a
	RTSV	20.4 ± 1.0b	32.9 ± 1.1b	43.8 ± 1.5b	53.3 ± 0.9 b
*O. glaberrima*—104545	Mock	28.8 ± 1.1a	48.3 ± 1.2a	68.2 ± 1.7a	87.0 ± 2.4a
	RTSV	25.2 ± 0.5b	39.6 ± 0.9b	58.3 ± 1.6b	73.5 ± 1.2b

1 *Mean ± standard error of mean based on three independent experiments. Values for mock- and RTSV-inoculated plants of the same rice genotype at the same days post-inoculation followed by different letters are significantly different by a t-test at the 95% confidence level*.

**Figure 1:**
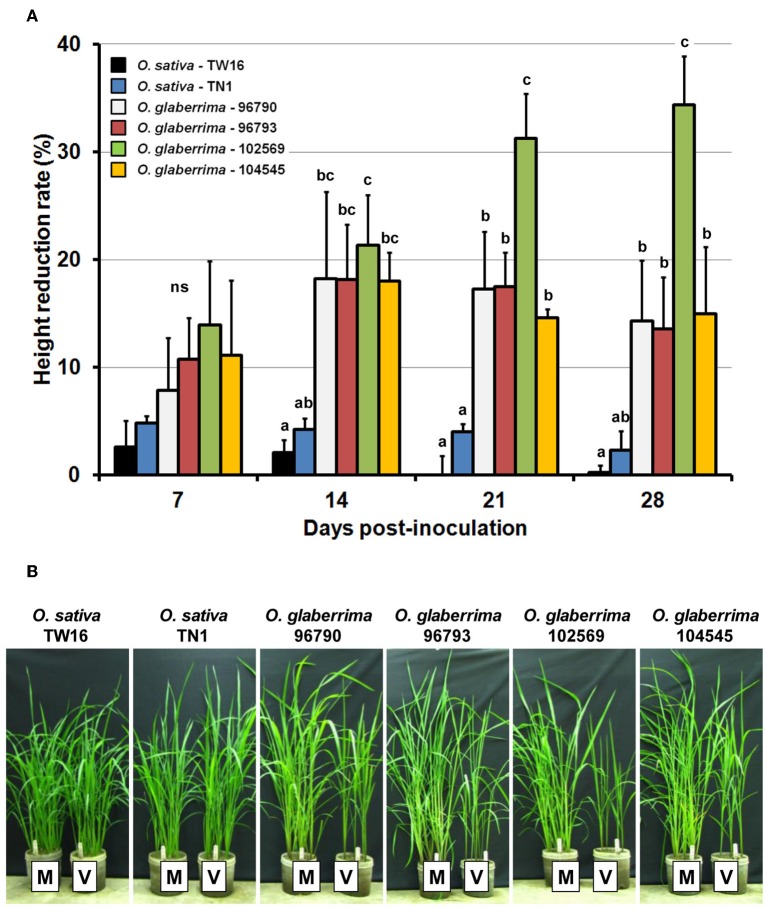
**Height reduction in *Oryza glaberrima* and *O. sativa* infected with *Rice tungro spherical virus* (RTSV)**. **(A)** Temporal changes in height reduction rates. Vertical bars and vertical lines indicate mean and standard error of mean, respectively, for the height reduction rate at the respective time points. Means and standard error of mean were based on three independent experiments (*n* = 5 per treatment and time point in one experiment). Bars at the same days post-inoculation (dpi) indicated by different letters are significantly different by the least significant difference test at the 95% confidence level. ns: not significantly different. **(B)** Plants of *O. glaberrima* and *O. sativa* at 28 dpi with RTSV. M, mock-inoculated plant; V, RTSV-inoculated plant.

The height reduction rates of the *O. sativa* and *O. glaberrima* plants were compared to examine whether the severity of stunting by RTSV infection was different among the plants. The height reduction rates of *O. glaberrima*-102569 were significantly higher than those of *O. sativa* TN1 and TW16 at 14 dpi and onwards, and they were also significantly higher than those of the other three *O. glaberrima* accessions at 21 and 28 dpi (Figure 1A, Supplementary Material 2). The reduction rates of *O. glaberrima*-96790, -96793, and -104545 were significantly higher than that of *O. sativa* TN1 only at 21 dpi. (Figure 1A, Supplementary Material 2). The height reduction rates of the four accessions of *O. glaberrima* were between approximately 8 and 14% at 7 dpi (Figure 1A, Supplementary Material 2). The height reduction rates for the four accessions of *O. glaberrima* increased to approximately 18–21% at 14 dpi, indicating that stunting of plants became more evident at 14 dpi. Stunting of *O. glaberrima*-102569 became more severe after 14 dpi, showing height reduction rates of approximately 31% at 21 dpi and 34% at 28 dpi (Figures 1A,B, Supplementary Material 2). In contrast, the height reduction rates of three other *O. glaberrima* accessions (96790, 96793, and 104545) decreased from approximately 18% at 14 dpi to approximately 14–15% at 28 dpi, indicating that stunting of these three *O. glaberrima* accessions became milder (Figures 1A,B, Supplementary Material 2). The height reduction rates for *O. sativa* TN1 and TW16 were between approximately −0.3 and 5% during the observation (Figure 1A, Supplementary Material 2), but the differences in height between the mock-inoculated *O. sativa* plants and the corresponding RTSV-infected plants were not statistically significant throughout the observation (Table 2). These results suggested that, unlike *O. sativa*, a majority of *O. glaberrima* accessions are vulnerable to RTSV, but the severity of stunting varies significantly among *O. glaberrima* accessions.

### Accumulation of RTSV in *O. glaberrima* and *O. sativa*

**Figure 2:**
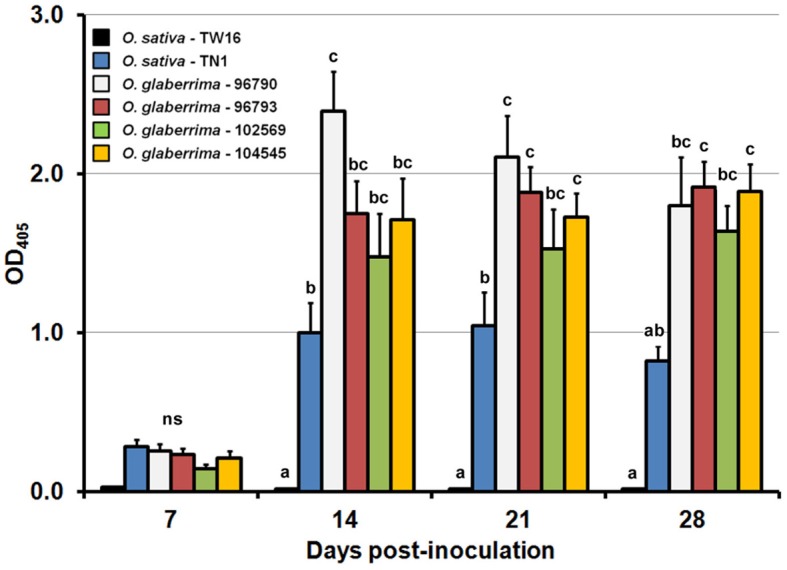
**Temporal changes in the accumulation of *Rice tungro spherical virus* in plants of *Oryza glaberrima* and *O. sativa***. Vertical bars and vertical lines indicate means and standard errors of mean, respectively, for the absorbance at 405 nm at the respective time points. Means and standard errors of mean were based on three independent experiments (*n* = 5 per treatment and time point in one experiment). Bars at the same days post-inoculation indicated by different letters are significantly different by the least significant difference test at the 95% confidence level. ns: not significantly different.

The accumulation of RTSV in the *O. glaberrima* and *O. sativa* plants was examined to see whether it is correlated with the height reduction rate. The levels of RTSV accumulation determined by ELISA in the four accessions of *O. glaberrima* and *O. sativa* increased rapidly until 14 dpi and then were maintained at similar levels or decreased slightly until 28 dpi (Figure 2, Supplementary Material 3). The accumulation of RTSV in *O. glaberrima*-96790 (OD_405_ of 2.40 ± 0.25) was significantly higher than that in the other three *O. glaberrima* accessions (1.48 ± 0.27–1.75 ± 0.21) at 14 dpi. The levels of RTSV accumulated in *O. glaberrima*-96793, -102569, and -104545 at a time point were not significantly different from one another throughout the observation. The levels of RTSV accumulated in *O. sativa* TN1 were significantly lower than those in at least one of the *O. glaberrima* accessions-96790, -96793, and 104545 at 14 dpi and onwards. The levels of RTSV accumulated in *O. glaberrima*-102569 were not significantly different from those in *O. sativa* TN1 throughout the observation, despite the fact that their height reduction rates were significantly different at 14 dpi and onwards. Accumulation of RTSV in *O. sativa* TW16 was not detected by ELISA. However, as in the previous studies by Encabo et al. (2009) and Satoh et al. (2013) showing detection of a very low level of RTSV in TW16 by RT-PCR, we assumed that most *O. sativa* TW16 plants inoculated with RTSV were infected since most *O. sativa* TN1 plants (susceptible control) inoculated at the same time were found to be infected with RTSV. The comparison between the height reduction rates (Figure 1, Supplementary Material 2) and the RTSV accumulation (Figure 2, Supplementary Material 3) indicates that the level of RTSV accumulation in plants is not correlated with the severity of stunting.

### Suppression of cell wall-related genes in *O. glaberrima* infected with RTSV

Temporal changes in expression of six cell wall-related genes were examined in *O. glaberrima*-96793 and -102569 and *O. sativa* TN1 to find cell wall-related genes associated with stunting of *O. glaberrima* caused by RTSV. The three accessions were selected since the severity of stunting by RTSV was significantly different from one another at 21 dpi (Figure 1, Supplementary Material 2). A genome-wide gene expression analysis in *O. sativa* TN1 and TW16 had been performed previously by Satoh et al. (2013). Genes for CESA5 and cellulose synthase A6 (CESA6), CSLA9 and cellulose synthase-like C7 (CSLC7), expansin 1, and α-expansin 15 precursor (expansin 15) were selected based on their expression patterns in *O. sativa* TN1 infected with RTSV. The previous microarray analysis by Satoh et al. (2013) showed that the expression 1) was activated for CESA5 and CSLC7 genes, 2) was suppressed for CSLA9 and expansin 15 genes, and 3) was not significantly changed for CESA6 and expansin 1 genes by RTSV infection between 6 and 15 dpi (Table 3).

**Table 3:** **Changes in expression of six cell wall-related genes in rice plants infected with *Rice dwarf virus* (RDV), *Rice stripe virus* (RSV), *Rice tungro bacilliform virus* (RTBV), and *Rice tungro spherical virus* (RTSV) examined by genome-wide microarray**.

**Gene for**	**Locus ID**	**Fold change in gene expression in**										
		***O. sativa* (Taichung Native 1) by**							***O. sativa* (Nipponbare) by**			
		**RTSV^1^ at**			**RTBV^2^ at**				**RSV^3^ at**			**RDV^4^ at**
		**6 dpi**	**9 dpi**	**15 dpi**	**6 dpi**	**9 dpi**	**12 dpi**	**18 dpi**	**6 dpi**	**9 dpi**	**12dpi**	**21 dpi**
Cellulose synthase A5 (CESA5)	LOC_Os03g62090	2.00	1.74	1.85	NS^5^	1.96	NS	NS	NS	NS	NS	NS
Cellulose synthase A6 (CESA6)	LOC_Os07g14850	NS	NS	NS	NS	NS	NS	NS	NS	NS	0.62	0.76
Cellulose synthase-like A9 (CSLA9)	LOC_Os06g42020	0.95	0.43	0.65	NS	0.30	0.56	0.44	NS	NS	NS	NE^6^
Cellulose synthase-like C7 (CSLC7)	LOC_Os05g43530	1.54	1.37	1.36	NS	NS	0.86	NS	NS	NS	NS	NS
α-Expansin 1 precursor	LOC_Os05g39990	NS	NS	NS	NS	0.10	0.13	0.22	NS	0.66	0.51	0.36
α-Expansin 15 precursor	LOC_Os02g51040	0.60	0.69	0.75	NS	0.32	NS	0.41	NS	NS	NS	0.56

1 *Data from Satoh et al. (2013)*.

2 *Unpublished data by K. Satoh*.

3 *Data from Satoh et al. (2010)*.

4 *Data with RDV strain S from Satoh et al. (2011)*.

5 *Change in expression not significant*.

6 *Expression not detected*.

**Figure 3:**
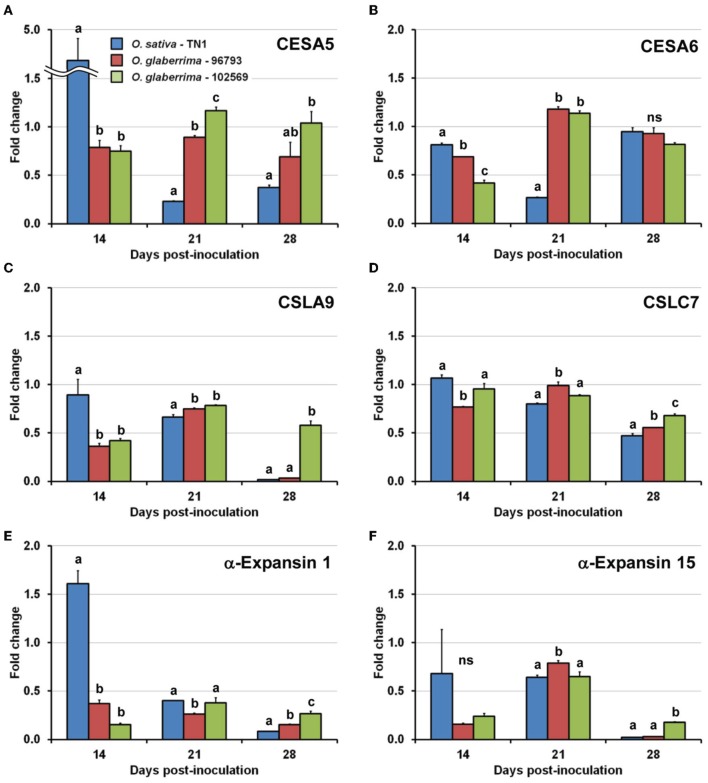
**Changes in expression of cell wall-related genes after infection with *Rice tungro spherical virus* (RTSV) in plants of *Oryza glaberrima* and *O. sativa* examined by real-time RT-PCR**. Vertical bars and vertical lines indicate means and standard errors of mean, respectively, for the fold changes in expression of genes for **(A)** cellulose synthase A5 (CESA5), **(B)** cellulose synthase A6 (CESA6), **(C)** cellulose synthase-like A9 (CSLA9), **(D)** cellulose synthase-like C7 (CSLC7), **(E)** α-expansin 1 precursor (α-Expansin 1), and **(F)** α-expansin 15 precursor (α-Expansin 15) between mock and RTSV-inoculated plants at the respective time points. Means and standard errors of mean were based on three replicated reactions. Bars at the same days post-inoculation indicated by different letters are significantly different by the least significant difference test at the 95% confidence level. ns: not significantly different.

At 14 dpi, the relative expression levels (fold changes) of four (CESA5, CESA6, CSLA9, and expansin 1genes) out of the six genes examined were significantly higher in *O. sativa* TN1 than in at least either *O. glaberrima*-96793 or -102569 (Figure 3). The expression of the CESA5 gene was up-regulated more than 4-fold by RTSV infection in *O. sativa* TN1 at 14 dpi, whereas the expression of the gene was found to be down-regulated slightly (approximately 0.7- to 0.8-fold) by RTSV infection in *O. glaberrima*-96793 and -102569 (Figure 3A). The expression of the expansin 1 gene was also up-regulated approximately 1.6-fold by RTSV infection in *O. sativa* TN1 at 14 dpi, whereas the gene was down-regulated to approximately 0.2- to 0.4-fold in both *O. glaberrima* accessions (Figure 3E). The expression of the genes for CESA6 and CSLA9 was down-regulated more significantly by RTSV infection in *O. glaberrima*-96793 and -10569 than in *O. sativa* TN1 at 14 dpi (Figures 3B,C).

At 21 dpi, the relative expression levels of five genes (CESA5, CESA6, CSLA9, CSLC7, and expansin 15 genes) were significantly higher in *O. glaberrima*-96793 than in *O. sativa* TN1 (Figure 3). The expression of all six genes was down-regulated to approximately 0.2- to 0.8-fold in *O. sativa* TN1 at 21 dpi (Figure 3). The expression of the genes for CESA5 and CESA6 was up-regulated slightly (approximately 1.1-to 1.2-fold) in *O. glaberrima*-102569 at 21 dpi (Figures 3A,B).

At 28 dpi, the relative expression levels of five genes (CESA5, CSLA9, CSLC7, expansin 1, and expansin 15 genes) were significantly higher in *O. glaberrima*-102569 than in *O. sativa* TN1 (Figure 3). The relative expression levels of four genes (CSLA9, CSLC7, expansin 1, and expansin 15 genes) were significantly higher in *O. glaberrima*-102569 than in *O. glaberrima*-97693 (Figure 3). The expression of all six genes was down-regulated in *O. sativa* TN1 (approximately 0.02- to 0.95-fold), and also in *O. glaberrima*-96793 (approximately 0.03- to 0.92-fold) at 28 dpi (Figure 3).

Overall, at 14 dpi, the relative expression levels of a majority of the six cell wall-related genes were significantly higher in *O. sativa* TN1 than in the two accessions of *O. glaberrima* which were stunted by RTSV infection. In contrast, at 28 dpi, the relative expression levels of a majority of the six genes were significantly higher in most severely stunted *O. glaberrima*-102569 than in *O. sativa* TN1 and *O. glaberrima*-96793.

## Discussion

RTSV has been considered as a latent virus since the accumulation of RTSV alone does not induce recognizable symptoms in most *O. sativa* genotypes. However, the lack of symptoms in *O. sativa* infected with RTSV might be due to defense mechanisms activated in *O. sativa*, not due to the lack of pathogenicity in RTSV. RTSV alone induces significant changes in the expression of diverse defense- and development-related genes in *O. sativa* (Encabo et al., 2009; Satoh et al., 2013). RTSV and RTBV synergistically enhance symptoms in *O. sativa* (Cabauatan et al., 1995). Along with these observations, the stunted growth of *O. glaberrima* plants by RTSV observed in Cabauatan et al. (1993) and this study suggests that, despite the potential pathogenicity of accumulating RTSV, most *O. sativa* genotypes may share a species-specific tolerance mechanism that prevents them from being stunted by RTSV. The accumulation of RTSV in *O. sativa* TN1 was significantly lower than that in three *O. glaberrima* accessions-96790, -96793, and -104545 at least at one time point during the observation (Figure 2, Supplementary Material 3). Therefore, alternatively, most *O. sativa* genotypes may operate a horizontal defense mechanism to suppress the accumulation of RTSV below the threshold level necessary to trigger a significant height reduction.

The severity of stunting (Figure 1, Supplementary Material 2) and the levels of RTSV accumulation (Figure 2, Supplementary Material 3) were significantly variable among the *O. glaberrima* accessions, but a clear relationship between the severity of stunting and the accumulation of RTSV was not observed. A lack of correlation between virus accumulation and severity of stunting was also the case with the interactions between *O. sativa* cultivar Nipponbare and three RDV strains (Satoh et al., 2011). Plants infected with RDV strain S were more severely stunted than those infected with RDV strain D84, yet the accumulation of the two RDV strains in the plants was not significantly different. Thus, the severity of stunting in the *Oryza* species might be associated with a virus-induced modification of gene expression decoupled from the level of virus accumulation, as was observed in the interaction of RTSV with *O. sativa* TN1 and TW16 (Satoh et al., 2013). For example, the expression of stress response-related genes such as those for TIFY transcription factors (Ye et al., 2009) and those for glutathione S-transferases was more activated in TW16, in which RTSV accumulated significantly less than in TN1, whereas the expression of development-related genes such as the homeobox gene family was more suppressed by RTSV in TW16.

Stunting of *O. sativa* plants by infection with RDV (Shimizu et al., 2007; Satoh et al., 2011) and RSV (Satoh et al., 2010) was accompanied by the suppression of cell wall-related genes such as those for cellulose synthase (-like), expansin, and extensin. The severity of stunting in *O. sativa* cultivar Nipponbare infected with three strains of RDV was associated with the suppression of dozens of cell wall-related genes (Satoh et al., 2011). Stunting of plants and impaired leaf growth were also induced by virus-induced silencing of cellulose synthase genes in *Nicotiana benthamiana* (Burton et al., 2000), by mutations in cellulose synthase genes in rice (Tanaka et al., 2003), and by artificial repression of expansin genes in *Arabidopsis thaliana* (Goh et al., 2012), and in rice (Choi et al., 2003). These observations suggest that suppression of particular cell wall-related genes in *O. glaberrima* after RTSV infection may have led to stunting of the plants. Among the expression patterns of the six cell wall-related genes in the *O. glaberrima* and *O. sativa* plants infected with RTSV (Figure 3), the evident activation of the CESA5 and the expansin 1 genes in *O. sativa* TN1 and the suppression of the two genes in the two accessions of *O. glaberrima* at 14 dpi appeared to be signature responses in the plants stunted (*O. glaberrima*-97693 and -102569) by RTSV infection and the plant not stunted (*O. sativa* TN1). In fact, the expression of the expansin 1 gene was consistently suppressed in *O. sativa* plants stunted by RDV, RSV, and RTBV, but not in *O. sativa* infected but not stunted by RTSV (Table 3). Thus, the gene for expansin 1 is likely to be the gene commonly associated with virus-induced stunting of *Oryza* species. Expansin loosens cell walls (Cosgrove, 1998), which may make plants more vulnerable to pathogens (Ding et al., 2008). Therefore, virus-induced stunting of *O. sativa* and *O. glaberrima* accompanying the suppression of the expansin 1 gene may be a general consequence of the defense response against viruses in *Oryza* species. Together with the suppression of the expansin 1 gene, more significant suppression of the CESA5, CESA6, and CSLA9 genes in *O. glaberrima*-96793 and -102569 than in *O. sativa* TN1 might be involved in the stunting of *O. glaberrima* by RTSV.

The results of this study suggested that stunting of *O. glaberrima* might be associated with the cell wall-related genes suppressed by RTSV. Genetic factors linked to the vulnerable reaction of *O. glaberrima* to RTSV can be accidentally transferred into *O. sativa* during the inter-specific hybridization intended for the improvement of Asian rice varieties. Therefore, characterization of the genetic factors underlying the vulnerability of *O. glaberrima* to RTSV is critical to avoid the accidental introduction of such factors into *O. sativa*.

### Conflict of interest statement

The authors declare that the research was conducted in the absence of any commercial or financial relationships that could be construed as a potential conflict of interest.

## Supplementary material

Supplementary Material 1 **Height and RTSV accumulation in 18 accessions of *O. glaberrima* infected with RTSV—See a separate file**.

Supplementary Material 2 **Temporary changes in height reduction rates in *O. sativa* (TW16, Taichung Native 1), and *O. glaberrima* (IRGC accession numbers 96790, 96793, 102569, and 104545)**.

Supplementary Material 3 **Temporary changes in accumulation of *Rice tungro spherical virus* in *O. sativa* (TW16, Taichung Native 1), and *O. glaberrima* (IRGC accession numbers 96790, 96793, 102569, and 104545) examined by an enzyme-linked immunosorbent assay**.
